# Are side effects necessary for antidepressant treatment?

**DOI:** 10.1192/j.eurpsy.2025.84

**Published:** 2025-08-26

**Authors:** S. Kasper

**Affiliations:** Department of Molecular Neuroscience, Medical University of Vienna, Vienna, Austria

## Abstract

**Abstract:**

The recent development of the socalled “psychedelics” reminds us that unfortunately some medications which we used in psychiatry have a large burden of side effects, like the anticholinerg side effects of the older tricyclic antidepressants as well as the extrapyramidal motoric side effects of socalled typical neuroleptics. These side effects were sometimes also related to the efficacy of these medications. Interestingly, it seems that the neglection of side effects is still an unresolved issue in clinical psychopharmacology, since there are researchers and clinicians who argue that the psychedelic experience induced with psychedelics are associated with therapeutic efficacy. Furthermore, studies in this field not even mention these side effects as such and argue, when confronted with the issue, that these are necessary for the therapeutic outcome. Even more so, there are researchers and clinicians who think that these side effects allow the patients to understand their unconscious, like in the early days of psychoanalysis. However, recent preclinical animal models demonstrated antidepressant-like behavioral effects and synaptic actions that are not only linked to the serotonergic activation (mainly via the 5HT2A receptor), but also via opioid and glutamatergic pathways which share neurobiological mechanisms of network reconfiguration likely by intracellular plasticity cascades. It seems to be important from my point of view to develop antidepressant medications devoid of the side effect of psychedelic experience in order to produce a safer, non-hallucinogenic medication that has therapeutic potential for depressed patients.

**Disclosure of Interest:**

S. Kasper Consultant of: In the past 3 years Dr Kasper served as a consultant or on advisory boards for Angelini, Biogen, Boehringer, Esai, Janssen, IQVIA, Mylan, Recordati, Rovi, and Schwabe; , Speakers bureau of: In the past 3 years Dr. Kasper served on speakers bureaus for Angelini, Aspen Farmaceutica S.A., Biogen, Janssen, Recordati, Schwabe, Servier, and Sothema.

